# Effects of single-dose and co-supplementation of vitamin D and omega-3 on metabolic profile in women with polycystic ovary syndrome: An RCT

**DOI:** 10.18502/ijrm.v21i7.13889

**Published:** 2023-08-23

**Authors:** Hadis Bahramian, Saeed Sherafatmanesh, Nasrin Asadi, Ali Bakhshi, Mohammad Hassan Eftekhari, Maryam Ekramzadeh6comma

**Affiliations:** ^1^Student Research Committee, School of Nutrition and Food Sciences, Shiraz University of Medical Sciences, Shiraz, Iran.; ^2^Nutrition and Food Security Research Center, Department of Nutrition, School of Public Health, Shahid Sadoughi University of Medical Sciences, Yazd, Iran.; ^3^Maternal-Fetal Medicine Research Center, Shiraz University of Medical Sciences, Shiraz, Iran.; ^4^Department of Clinical Biochemistry, Faculty of Medicine, Shahid Sadoughi University of Medical Sciences, Yazd, Iran.; ^5^Department of Clinical Nutrition, School of Nutrition and Food Sciences, Shiraz University of Medical Sciences, Shiraz, Iran.; ^6^Nutrition Research Center, Department of Clinical Nutrition, School of Nutrition and Food Sciences, Shiraz University of Medical Sciences, Shiraz, Iran.; ^7^Division of Nephrology and Hypertension, Lundquist Institute, Harbor-UCLA Medical Center, Torrance, CA 90502, USA.

**Keywords:** Polycystic ovary syndrome, Vitamin D, Omega-3 fatty acid, Insulin, Sex hormone-binding globulin.

## Abstract

**Background:**

Polycystic ovary syndrome (PCOS) is a heterogeneous medical condition with a cluster of metabolic and endocrine disorders including dyslipidemia, insulin resistance, and hyperandrogenism.

**Objective:**

The present study aimed to determine the effects of single-dose and co-supplementation of vitamin D (vit D) and omega-3 (O3) on anthropometric and several biochemical factors in women with PCOS.

**Materials and Methods:**

In this double-blind, randomized clinical trial, 80 PCOS women referred to Shahid Motahhari Clinic, Shiraz, Iran, from April to October 2017 were studied in 4 groups (n = 20/each) for 8 wk. The placebo group received the placebo capsule (paraffin oil); 1 weekly and 2/daily; the vit D group received vit D (50,000 IU/weekly) + 2 placebo capsules daily, O3 group, 2, O3 capsules daily + 1 placebo capsule weekly, and vit D + O3 (50000 IU/weekly vit D + 2, O3 capsules daily). Before and after 8 wk of intervention, height, weight, body mass index, waist circumference, triglycerides, total cholesterol (TC), high-density lipoprotein cholesterol, low-density lipoprotein cholesterol, fasting blood sugar, homeostasis model of insulin resistance index, and sex hormone binding globulin were compared between groups.

**Results:**

The significant reduction was detected in serum triglyceride (p = 0.002), TC (p = 0.04), fasting blood sugar (p = 0.02), insulin (p = 0.001), and homeostasis model of insulin resistance index (p = 0.001) concentrations in all vit D, O3, and vit D + O3 supplemented groups compared to the placebo group. Furthermore, in comparison with the placebo group, a significant increase was observed in serum sex hormone binding globulin levels after vit D, O3, and vit D + O3 treatments. Nevertheless, no significant changes were observed in serum high-density lipoprotein cholesterol, low-density lipoprotein cholesterol, and anthropometric indices in all treated participants.

**Conclusion:**

The current study indicated that single dose and co-supplementation of vit D and O3 for 8 wk was associated with beneficial effects on serum triglyceride, TC, insulin, and sex hormone binding globulin concentrations among women suffering from PCOS.

## 1. Introduction

Polycystic ovary syndrome (PCOS) is one of the most common endocrine ailment, affecting 5-10% of females worldwide during their reproductive period (1). It is the main cause of infertility and unfavorable pregnancy consequences caused by the dysfunctional phase of follicular maturation and ovulation, multicystic condition of ovaries, and hyperandrogenism (1). Furthermore, PCOS along with a group of risk factors is related to the development of cardiovascular disease and type 2 diabetes including insulin resistance, hyperglycemia, lipid profile abnormalities, and abdominal obesity (2).

Although the etiology of PCOS remains unclear, the underlying preventable and controllable mechanisms like obesity, hyperinsulinemia, and hyperandrogenism have provided new insights into the pathogenesis of this disease (3). It has been documented that individuals with PCOS can benefit from a combination of different nutrients, as well as improve their overall nutritional status (4). Hence, many researchers have focused on nutrition-based lifestyle modification interventions in women with PCOS as a global necessity.

Vitamin D (vit D) deficiency is one of the well-known health problems observed in nearly 67-85% of women with PCOS, worldwide (5). The effects of vit D are mediated by both genetic and cellular mechanisms. It controls gene transcription via the intracellular distribution of nuclear vit D receptors in several tissues, specifically the ovaries (6). There is accumulating evidence that shows that serum vit D levels may be negatively implicated in PCOS symptoms, including obesity, hyperlipidemia, insulin resistance, hyperandrogenism, diminished sex hormone binding globulin (SHBG) production, and higher risks of cardiovascular disease, and abortion (7, 8). Also, it has been reported that vit D deficiency may contribute to the dysregulation of calcium metabolism, which in turn led to advanced follicular arrest and menstrual disturbances in women with PCOS (9).

Moreover, it has been documented that the consumption of unsaturated fat-rich foods may result in the improvement of PCOS metabolic impairments (10). As a bioactive anti-inflammatory agent, it has been confirmed that omega-3 (O3) supplementation can exhibit beneficial effects in improving parameters such as fasting glucose, insulin resistance, triglyceride level, and ovulation function (11). Furthermore, this dietary supplement may be used for ameliorating insulin sensitivity by reducing the generation of inflammatory cytokines including tumor necrosis factor alpha and interleukin 6 (12).

Although the efficacy of single supplements (vit D and O3) has been evaluated in previous studies, the synergistic effects of both supplements in attenuating lipid and glycemic profiles have not been investigated in PCOS cases till now. Therefore, the present study aimed to determine the effects of single-dose and co-supplementation of vit D and O3 for the management of PCOS complications.

## 2. Materials and Methods

### Study design and participants

This double-blind, randomized clinical trial was conducted on 80 PCOS women. An established diagnosis of PCOS based on Rotterdam diagnostic criteria and vit D deficiency (25-hydroxyvitamin D ≤ 20 nmol/L) was recruited after screening 107 participants (aged 18-45 yr) from outpatients visiting, 18.5 ≤ body mass index (BMI) ≤ 40 referred to Shahid Motahhari Clinic, Shiraz, Iran, from April to October 2017.

The exclusion criteria were listed as follows: having a background of chronic illnesses such as cancer, heart disease, diabetes, stroke, fibromyalgia, kidney or liver defects, having a history of food and drug allergies, starting the drug or surgical therapy for clinical symptoms associated with PCOS except for oral contraceptive pills, smoking or any drug addiction, pregnancy and lactation, being on a special diet in the last year, using any dietary supplement, having oral or injectable nutritional supplements containing vit D in the last 3 months, consuming nutritional supplements containing fish oil or O3 fatty acids in the last 3 months, having fish in the diet more than 3 servings per week during last 3 months, background of serious side effects or signs of poisoning from the study's supplements, and failure to follow the research protocol.

### Sample size

With respect to the type I error of 5% (α = 0.05), and type II error of 20% from the prior investigation in PCOS cases (13), the sample size was calculated based on the following formula: 


$$n=\frac{2\delta^{2}{\left(Z_{1-\frac{\alpha }{2}}+Z_{1-\beta }\right)}^{2}}{{\left({µ}_{1}-{µ}_{2}\right)}^{2}}$$

### Randomization, blinding, and interventions

Computer-generated randomization was used to complete the randomization process, and a qualified clinician carried out the randomized allocation procedure and allocated individuals as 1: 1: 1: 1 to each of the study groups. The randomization sequence concealment continued until the end of the intervention course. The researcher and participants were blinded.

The participants were randomly divided into 4 groups (n = 20/each) for 8 wk by block randomization with a fixed block size of 4 for 8 wk as follows:

Vit D group: received 1 vit D capsule (Zahravi Pharmaceutical Company, Tehran, Iran) (50,000 IU/weekly) + 2 placebo capsules (paraffin oil; daily).

O3 group: received 2 O3 capsules (Zahravi Pharmaceutical Company, Tehran, Iran) daily (each one contained 360 mg eicosapentaenoic acid and 240 mg docosahexaenoic acid) + 1 placebo capsule (paraffin oil; weekly).

Vit D + O3 group: received 1 vit D capsule (50,000 IU/weekly) + 2 O3 capsules daily (each one contained 360 mg eicosapentaenoic acid and 240 mg docosahexaenoic acid).

Placebo group: received 1 placebo capsule (paraffin oil; weekly) + 2 placebo capsules (paraffin oil; daily).

The amount of vit D and O3 supplements were according to the prior studies that showed an improvement in biochemical factors with the fewest side effects (13, 14). All vit D, O3, and placebo capsules were prepared by Zahravi Pharmaceutical Company, Tehran, Iran. The placebo capsules had the same size, shape, and color in comparison to capsules containing vit D or O3. Each week, telephone conversations were used to evaluate the subjects' compliance with confirmation of taking the capsules. Furthermore, cases were visited every 2 wk during the study period for accurate follow-up assessment. Participants' physical activity was measured by filling out an international physical activity questionnaire (15). Additionally, we requested all participants not to modify their level of physical activity, dietary habits, and hours of exposure to sunlight during the study phase.

### Outcomes

Measurements were performed by trained investigators before and after 8 wk of intervention, including height, weight, BMI, waist circumference (WC), triglycerides (TG), total cholesterol (TC), high-density lipoprotein cholesterol (HDL), low-density lipoprotein cholesterol (LDL), fasting blood sugar (FBS), homeostasis model of insulin resistance index (HOMA-IR), serum insulin level, physical activity, and SHBG were compared between groups. All laboratory procedures were carried out following accepted laboratory practices under the supervision of competent professionals in the Nutrition and Food Science School's lab at Shiraz University of Medical Sciences, Shiraz, Iran.

### Ethical considerations

All participants were informed about the study objectives and asked to sign the written consent form. The procedure of this study was conducted according to the Declaration of Helsinki. It was also approved by the Ethics Committee on Human Experimentation of Shiraz University of Medical Sciences, Shiraz, Iran (Code: IR.SUMS.REC.1396.103). Furthermore, this trial was registered at the Iranian Registry of Clinical Trials and has been updated on September 19, 2022.

### Statistical analysis

Statistical analysis was done using the SPSS software (Statistical Package for the Social Sciences, version 22.0, SPSS Inc, Chicago, Illinois, USA). First, the normal distribution of the data was assessed by Kolmogorov-Smirnov test. The Kruskal-Wallis test was used to compare the 4 groups regarding mean differences. Then, the Mann-Whitney U test was employed for comparing the means of quantitative data between groups. The Wilcoxon test was used to compare the changes in variables before and after the intervention in each group. All the differences were considered statistically significant at p ≤ 0.05.

## 3. Results

**Figure 1:**
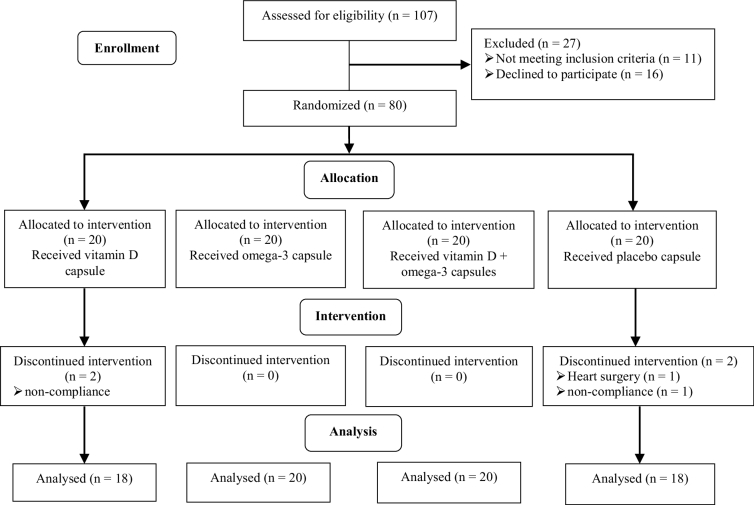
CONSORT flow diagram of trial.

Table I summarizes the general characteristics of the research participants. At the beginning of the experiment, no statistically significant differences were identified among the 4 study groups, as shown in the table. 4 people were dropped from the research during the intervention phase because of non-compliance or heart surgery, and 76 individuals completed the trial (Figure 1). No serious side effects were detected in all study groups.

### Anthropometric indices

By the end of the study, no significant differences were observed between all study groups in terms of height, body weight, BMI, WC, and physical activity (Table II).

The mean values of anthropometric and biochemical parameters in each study group, before and after the study, have been presented in (Table III). After 8 wk intervention, the WC showed a significant decrease in vit D and O3 groups in comparison with the baseline levels. Also, compared to the values at the beginning of the study, a statistically significant reduction of WC was observed in vit D + O3 treated participants. However, at the end of the study, no significant difference was found in terms of body weight and BMI parameters in all the study groups compared to the baseline period.

### Serum lipid profile

The effect of study treatments on the serum biochemical indices has been shown in (Table II). By the end of the experiment, no statistically significant difference was observed between any of the study groups regarding the serum HDL_C and LDL_C levels. Meanwhile, vit D, O3, and vit D + O3 treatments resulted in significantly lower TG levels as compared to cases treated with placebo [(p = 0.02), (p = 0.004), and (p = 0.004), respectively]. Furthermore, compared to the placebo group, all the vit D, O3, and vit D + O3 treatments showed a significant decrease in TC concentration after the study [(p = 0.04), (p = 0.01), and (p = 0.008), respectively].

In addition, compared to the baseline course, serum TC was remarkably decreased in vit D, O3, and vit D + O3 supplemented groups. Nevertheless, there were no significant changes in other serum lipid parameters at the end of the study when compared to the baseline (Table III).

### FBS, serum insulin, and HOMA-IR parameters

By the end of the study, vit D, O3, and vit D + O3 interventions led to a significantly lower FBS level in comparison with the individuals treated with placebo [(p = 0.01), (p = 0.03), and (p = 0.007), respectively]. Moreover, a lower concentration of serum insulin was detected in the vit D, O3, and vit D + O3 groups in comparison with the amounts in the placebo group [(p = 0.002), (p = 0.002), and (p = 0.001), respectively]. As well, compared to the placebo group, interventions with vit D, O3, and vit D + O3 provided significantly lower HOMA-IR levels [(p = 0.002), (p = 0.007), and (p = 0.001), respectively]. In this regard, compared to the vit D and O3 treatments, the oral co-supplementation with vit D + O3 was accompanied by a synergistically significant reduction in serum FBS [(p = 0.04), (p = 0.03), respectively] and HOMA-IR [(p = 0.02), (p = 0.01), respectively] levels (Table II).

Moreover, in comparison with the baseline period, serum insulin was notably decreased in the vit D, O3, and vit D + O3 treated groups when compared to the baseline. Furthermore, a statistically significant reduction was seen regarding the HOMA-IR level in the vit D, O3, and vit D + O3 supplemented groups when compared to the amounts at the beginning of the experiment (Table III).

### Serum SHBG hormone

In comparison to the placebo group, all vit D, O3, and vit D + O3 treatments showed a significant increase in SHBG concentration after the study [(p = 0.001), (p = 0.02), and (p = 0.02), respectively] (Table II).

Besides, there were notable enhancements in the SHBG parameter for the vit D, O3, and vit D + O3 groups compared with the values at the beginning of the trial (Table III).

**Table 1:** The participants' general characteristics and biochemical profiles


**Parameters**	**Vitamin D (n = 18)**	**Omega-3 (n = 20)**	**Vitamin D + Omega-3 (n = 20)**	**Placebo (n = 18)**	**P-value**
**Age (Yr)**	23.60 ± 3.42	22.29 ± 3.63	24.62 ± 3.11	22.35 ± 3.12	0.14
**Height (cm)**	161.33 ± 0.04	162.61 ± 0.05	160.38 ± 0.04	161.59 ± 0.06	0.93
**Weight (Kg)**	66.56 ± 22.22	66.71 ± 15.72	63.18 ± 6.78	65.30 ± 19.66	0.79
**BMI (kg/m^2^)**	25.47 ± 8.08	25.36 ± 4.97	24.85 ± 2.62	25.49 ± 6.35	0.81
**WC (cm)**	91.26 ± 18.54	89.00 ± 12.33	90.43 ± 6.59	90.05 ± 15.50	1.00
Physical activity (Met*min/wk)	924.4 ± 92.7	917.4 ± 73.42	918.9 ± 87.23	933.4 ± 89.01	0.07
**TG (mg/dl)**	119.66 ± 81.28	112.94 ± 30.93	113.87 ± 30.11	118.50 ± 40.68	0. 28
**TC (mg/dl)**	174.53 ± 29.60	161.11 ± 34.9	168.87 ± 28.54	169.78 ± 28.31	0.07
**HDL_C (mg/dl)**	31.04 ± 4.69	32.00 ± 6.47	33.50 ± 3.26	33.61 ± 3.75	0.55
**LDL_C (mg/dl)**	99.66 ± 32.97	103.64 ± 23.86	99.31 ± 33.05	96.37 ± 22.42	0.11
**FBS (mg/dl)**	105.23 ± 7.08	109.81 ± 5.21	106.83 ± 9.44	102.17 ± 6.57	0.13
**Serum insulin (mIU/L)**	36.01 ± 15.88	38.79 ± 25.55	40.14 ± 13.46	40.55 ± 5.12	0.47
**HOMA-IR**	8.80 ± 3.22	7.19 ± 4.64	7.94 ± 2.36	9.63 ± 1.25	0.08
**SHBG (ng/dl)**	119.08 ± 50.10	114.62 ± 74.96	109.37 ± 33.24	113.21 ± 55.94	0.39
Data expressed as Mean ± SD. Kruskal-Wallis test. BMI: Body mass index, WC: Waist circumference, TG: Triglyceride, TC: Total cholesterol, HDL_C: High-density lipoprotein cholesterol, LDL_C: Low-density lipoprotein cholesterol, FBS: Fasting blood sugar, HOMA-IR: Homeostatic model assessment of insulin resistance, SHBG: Sex hormone binding globulin					

**Table 2:** The participants' values of anthropometric and biochemical parameters after the study


**Parameters**	**Vitamin D (n = 18)**	**Omega-3 (n = 20)**	**Vitamin D + Omega-3 (n = 20)**	**Placebo (n = 18)**	**P-value**
**Height (cm)**	161.33 ± 0.04	162.61 ± 0.05	160.38 ± 0.04	161.59 ± 0.06	0.93
**Weight (Kg)**	66.34 ± 22.39 ${}^{\text{a}}$	66.74 ± 14.86 ${}^{\text{a}}$	62.86 ± 6.41 ${}^{\text{a}}$	65.14 ± 19.17 ${}^{\text{a}}$	0.84
**BMI (kg/m^2^)**	25.39 ± 8.14 ${}^{\text{a}}$	25.21 ± 4.72 ${}^{\text{a}}$	24.73 ± 2.48 ${}^{\text{a}}$	25.38 ± 6.09 ${}^{\text{a}}$	0.84
**WC (cm)**	89.73 ± 19.23 ${}^{\text{a}}$	87.52 ± 10.83 ${}^{\text{a}}$	89.71 ± 6.27 ${}^{\text{a}}$	91.47 ± 15.28 ${}^{\text{a}}$	0.12
Physical activity (Met*min/wk)	925.48 ± 85.2	917.12 ± 72.54	915.32 ± 39.43	934.02 ± 84.33	0.08
**TG (mg/dl)**	111.86 ± 69.00 ${}^{\text{a}}$	97.58 ± 24.15 ${}^{\text{a}}$	96.06 ± 28.74 ${}^{\text{a}}$	126.14 ± 32.32 ${}^{\text{b}}$	0.002
**TC (mg/dl)**	158.06 ± 24.50 ${}^{\text{a}}$	133.64 ± 37.93 ${}^{\text{b}}$	129.93 ± 27.22 ${}^{\text{b}}$	178.64 ± 32.78 ${}^{\text{c}}$	0.04
**HDL_C (mg/dl)**	32.53 ± 5.86 ${}^{\text{a}}$	32.48 ± 6.38 ${}^{\text{a}}$	34.06 ± 5.13 ${}^{\text{a}}$	33.22 ± 6.40 ${}^{\text{a}}$	0.18
**LDL_C (mg/dl)**	92.33 ± 23.92 ${}^{\text{a}}$	95.76 ± 25.03 ${}^{\text{a}}$	92.50 ± 19.31 ${}^{\text{a}}$	92.14 ± 27.51 ${}^{\text{a}}$	0.13
**FBS (mg/dl)**	98.47 ± 6.86 ${}^{\text{a}}$	100.02 ± 9.32 ${}^{\text{a}}$	94.23 ± 8.98	107.15 ± 6.64 ${}^{\text{c}}$	0.02
**Serum insulin (mIU/L)**	22.26 ± 6.34 ${}^{\text{a}}$	21.34 ± 2.34 ${}^{\text{ab}}$	20.98 ± 6.46 ${}^{\text{b}}$	38.62 ± 17.69 ${}^{\text{c}}$	0.001
**HOMA - IR**	4.82 ± 3.55 ${}^{\text{a}}$	4.88 ± 1.38 ${}^{\text{a}}$	4.01 ± 1.50 ${}^{\text{b}}$	9.36 ± 1.47 ${}^{\text{c}}$	0.001
**SHBG (ng/dl)**	136.87 ± 98.96 ${}^{\text{a}}$	129.25 ± 102.10 ${}^{\text{b}}$	144.82 ± 90.55 ${}^{\text{a}}$	104.24 ± 63.10 ${}^{\text{c}}$	0.004
Data expressed as Mean ± SD. Obtained from the Kruskal-Wallis test, ${}^{\text{a},\text{b},\text{c}}$ Obtained from the Mann-Whitney U test, BMI: Body mass index, WC: Waist circumference, TG: Triglyceride, TC: Total cholesterol, HDL_C: High-density lipoprotein cholesterol, LDL_C: Low-density lipoprotein cholesterol, FBS: Fasting blood sugar, HOMA-IR: Homeostatic model assessment of insulin resistance, SHBG: Sex hormone binding globulin					

**Table 3:** The participants' values of anthropometric and biochemical parameters before and after the study


	**Vitamin D (n = 18)**			**Omega-3 (n = 20)**			**Vitamin D + Omega-3 (n = 20)**			**Placebo (n = 18)**		
**Parameters**	**Before**	**After**	**P-value**	**Before**	**After**	**P-value**	**Before**	**After**	**P-value**	**Before**	**After**	**P-value**
	**Weight (Kg)**	66.56 ± 22.22	66.34 ± 22.39	0.55	66.71 ± 15.72	66.74 ± 14.86	0.34	63.18 ± 6.78	62.86 ± 6.41	0.16	65.30 ± 19.66	65.14 ± 19.17	0.28
**BMI (kg/m^2^)**	25.47 ± 8.08	25.39 ± 8.14	0.64	25.36 ± 4.97	25.21 ± 4.72	0.14	24.85 ± 2.62	24.73 ± 2.48	0.06	25.49 ± 6.35	25.38 ± 6.09	0.36
**WC (cm)**	91.26 ± 18.54	89.73 ± 19.23	0.005	89.00 ± 12.33	87.52 ± 10.83	0.04	90.43 ± 6.59	89.71 ± 6.27	0.006	90.05 ± 15.50	91.47 ± 15.28	0.20
**TG (mg/dl)**	119.66 ± 81.28	111.86 ± 69.00	0.17	112.94 ± 30.93	97.58 ± 24.15	0.11	113.87 ± 30.11	96.06 ± 28.74	0.10	118.50 ± 40.68	126.14 ± 32.32	0.21
**TC (mg/dl)**	174.53 ± 29.60	158.06 ± 24.50	0.04	161.11 ± 34.9	133.64 ± 37.93	0.02	168.87 ± 28.54	129.93 ± 27.22	0.01	169.78 ± 28.31	178.64 ± 32.78	0.39
**HDL_C (mg/dl)**	31.04 ± 4.69	32.53 ± 5.86	0.38	32.00 ± 6.47	32.48 ± 6.38	0.90	33.50 ± 3.26	34.06 ± 5.13	0.19	33.61 ± 3.75	33.22 ± 6.40	0.08
**LDL_C (mg/dl)**	99.66 ± 32.97	92.33 ± 23.92	0.21	103.64 ± 23.86	95.76 ± 25.03	0.17	99.31 ± 33.05	92.50 ± 19.31	0.21	96.37 ± 22.42	92.14 ± 27.51	0.26
**FBS (mg/dl)**	105.23 ± 7.08	98.47 ± 6.86	0.09	109.81 ± 5.21	100.02 ± 9.32	0.06	106.83 ± 9.44	94.23 ± 8.98	0.06	102.17 ± 6.57	107.15 ± 6.64	0.12
**Serum insulin** **(mIU/L)**	36.01 ± 15.88	22.26 ± 6.34	0.004	38.79 ± 25.55	21.34 ± 2.34	0.01	40.14 ± 13.46	20.98 ± 6.46	0.002	40.55 ± 5.12	38.62 ± 17.69	0.13
**HOMA - IR**	8.80 ± 3.22	4.82 ± 3.55	0.03	7.19 ± 4.64	4.88 ± 1.38	0.04	7.94 ± 2.36	4.01 ± 1.50	0.04	9.63 ± 1.25	9.36 ± 1.47	0.29
**SHBG (ng/dl)**	119.08 ± 50.10	136.87 ± 98.96	0.03	114.62 ± 74.96	129.25 ± 102.10	0.01	109.37 ± 33.24	144.82 ± 90.55	0.007	113.21 ± 55.94	104.24 ± 63.10	0.51
Data expressed as Mean ± SD. Wilcoxon test. BMI: Body mass index, WC: Waist circumference, TG: Triglyceride, TC: Total cholesterol, HDL_C: High-density lipoprotein cholesterol, LDL_C: Low-density lipoprotein cholesterol, FBS: Fasting blood sugar, HOMA-IR: Homeostatic model assessment of insulin resistance, SHBG: Sex hormone binding globulin												

## 4. Discussion

To the best of our knowledge, this study is a worthwhile effort to investigate the effects of single-dose and co-supplementation of vit D and O3 on anthropometric factors, lipid and glycemic profile, and SHBG status in women with PCOS.

PCOS is known as a metabolic disorder closely associated with hyperglycemia, insulin resistance, and dyslipidemia (2). The current study revealed that a single dose and co-supplementation of vit D and O3 for 8 wk resulted in improved HOMA-IR and serum TG, TC, FBS, insulin, and SHBG concentrations in PCOS participants.

Several studies have been conducted to investigate the occurrence of vit D deficiency in PCOS and its consequences on lipid and glycemic disorders (16, 17). It has been reported that inadequate vit D status would dramatically decrease vitamin D receptor activity, which in turn would result in higher circulating cholesterol levels through insulin-induced gene 2/sterol regulatory element-binding protein-2/3-hydroxy-3-methylglutaryl-CoA reductase-dependent signaling pathways (18). According to previous experiments, the effect of vit D on blood lipids may be due to the inhibition of parathyroid hormone (PTH) secretion, as elevated PTH has been shown to have a positive association with higher WC (19), suppressed lipolysis (20), and insulin resistance (21). Lower glucose uptake, as well as an increase in body fat accumulation, may result in ovarian and adrenal hyperandrogenism (22). Hypovitaminosis D may adversely contribute to hyperandrogenism through 2 different mechanisms: (1) upregulation of androgen synthesis in theca cells due to stimulation of the luteinizing hormone; (2) inhibition of SHBG production in the liver (23). Vit D may increase insulin secretion and stimulate glucose transfer via activation of the genes related to insulin receptor synthesis and production of insulin-regulated glucose transporter type-4 in the cell membrane (24). Moreover, this vitamin increases the production of the peroxisome proliferator-activated receptor gamma gene, which in turn enhances insulin sensitivity and fatty acid metabolism (24). Meanwhile, it has been reported in previous trials that adding O3 to vit D supplementation might enhance its beneficial impact on the metabolic profile of PCOS individuals (25). Several mechanisms have been proposed for the effect of O3 on glycemic indices and serum lipids, such as: (1) activation of AMP-activated protein kinase in the liver, white adipose tissues, and muscles which can reduce lipogenesis, improve the oxidation of fatty acids, and also promote glucose transfer into the cells, (2) O3 may increase insulin sensitivity and decrease adipocyte proliferation due to its multiple anti-inflammatory properties (26). In one study, the researchers found O3 as an effective agent in raising SHBG in non-obese PCOS women after 6-month therapy (27). Furthermore, several experiments have been conducted regarding the effects of vit D and O3 co-supplementation on different serum biochemical indices in other diseases. Consistent with the current findings, in a 6-wk investigation regarding the benefits of taking 50,000 IU of vit D every 2 wk and 1000 mg of O3 twice daily in women with gestational diabetes, it was found that co-supplementation with vit D and O3 led to an improvement in terms of glycemic regulation and triglyceride levels while having little effect on other serum lipids (28). Similar to the results of the present study, Gurol et al. (29) reported that vit D and O3 co-supplementation could be synergistically effective in lowering FBS concentration in pancreatic islets in transplanted rats. Additionally, another trial study investigated the impact of combined therapy with O3 (4-10 gr of fish oil/daily) and vit D (2000 IU/daily) on serum lipid profiles in individuals with subclinical arteriosclerosis. After 18 months follow-up, they found that the participants had lower TC, TG, and LDL-C levels, as well as a higher HDL-C concentration. The fact that their trial used a higher dose of O3 and vit D along with a longer study period may account for the inconsistency between LDL-C and HDL-C outcomes in the aforementioned study and the present experiment (30).

Our results should shed light while considering the main limitations. The intervention period appears to be insufficient to detect changes in other study indices, such as anthropometric parameters. As a result of limited funding, other important biochemical parameters related to the PCOS cases, such as androstenedione, dehydroepiandrosterone sulfate, PTH, and Ca^2+^, were not assessed. Future investigations are thus required to assess other particular indicators that may elucidate the advantageous underlying processes relating to the benefits of supplementation with vit D and O3 on PCOS complications.

## 5. Conclusion

Findings of the current study indicated that a single dose and co-supplementation of vit D and O3 for 8 wk was associated with beneficial effects on serum TG, TC, insulin, and SHBG concentrations. A positive synergistic effect of vit D and O3 co-administration was found regarding glycemic control and insulin resistance (FBS and HOMA-IR levels) among women suffering from PCOS.

##  Conflict of Interest

The authors declare that there is no conflict of interest.
